# Supplementary material for: Progressive remote memory decline coincides with parvalbumin interneuron hyperexcitability and enhanced inhibition of cortical engram cells in a mouse model of Alzheimer’s disease
Source: eLife. 2025 Nov 21;14:RP106866. doi: 10.7554/eLife.106866 (PMC12638044; doi:10.7554/eLife.106866)
Supplement: Supplementary file 1. [file elife-106866-supp1.docx]

**Progressive remote memory decline coincides with parvalbumin interneuron hyperexcitability and enhanced inhibition of cortical engram cells in a mouse model of Alzheimer’s disease**

Julia J. van Adrichem^1^, Rolinka J. van der Loo^1^, Romina Ambrosini Defendi^1^, August B. Smit^1^, Michel C. van den Oever^1, *^, Ronald E. van Kesteren^1, *^

^1^Department of Molecular and Cellular Neurobiology, Center for Neurogenomics and Cognitive Research, Amsterdam Neuroscience, Vrije Universiteit Amsterdam, Amsterdam, the Netherlands

*Shared senior authors

Correspondence:

R.E. van Kesteren: [ronald.van.kesteren@vu.nl](mailto:ronald.van.kesteren@vu.nl)

M.C. van den Oever: [michel.vanden.oever@vu.nl](mailto:michel.vanden.oever@vu.nl)

**Supplementary tables**

Supplemental tables

| **Supplementary Table 1A. Passive and active membrane properties of PV interneurons in the mPFC at 16 weeks** | | | |
| --- | --- | --- | --- |
|  | **PV interneurons** | | |
|  | **PV-Cre-tdTomato** | **APP/PS1-PV-Cre-tdTomato** | ***p-value*** |
| **Properties** | (mean ± SEM) | (mean ± SEM) | (Student’s *t*-test) |
| **Passive properties** | | | |
| RMP (mV) | -71.6 ± 0.88 | -71.1±1.04 | *0.98* |
| Rheobase (pA) | 258.1±16.60 | 256.3±14.57 | *0.93* |
| Current threshold (mV) | -30.7±1.17 | -32.1±0.71 | *0.3* |
| Input resistance (Mohm) | 94.2±3.48 | 93.4±5.44 | *0.50* |
| Membrane capacitance (pF) | 129.7±9.23 | 99.7±4.45 | **0.010* |
| Sag ratio | 0.062±0.007 | 0.063±0.004 | *0.90* |
| Time constant (ms) | 5.74±0.39 | 5.46±0.25 | *0.55* |
| **Active properties** | | | |
| AP amplitude (mV) | 53.4±1.19 | 51.9±0.98 | *0.34* |
| AP half-width (msec) | 0.38±0.012 | 0.40±0.010 | *0.38* |
| AHP (mV) | -18.3±0.48 | -17.6±0.45 | *0.32* |
| AHP time to peak (ms) | 1.28±0.070 | 1.31±0.045 | *0.24* |
| dv/dtmin (mV/ms) | -182.1±8.14 | -167.6±5.57 | *0.15* |
| dv/dtmax (mV/ms) | 284.5±9.61 | 265.80±6.33 | *0.11* |
| Interspike interval (ms) | 16.4±0.71 | 17.0±0.83 | *0.61* |
| Adaptation ratio | 1.02±0.031 | 1.09±0.063 | *0.72* |

| **Supplementary Table 1B. Passive and active membrane properties of PYR neurons in the mPFC at 16 weeks** | | | |
| --- | --- | --- | --- |
|  | **PYR neurons** | | |
|  | **PV-Cre-tdTomato** | **APP/PS1-PV-Cre-tdTomato** | ***p-value*** |
| **Properties** | (mean ± SEM) | (mean ± SEM) | v(Student’s *t*-test) |
| **Passive properties** | | | |
| RMP (mV) | -65.3 ± 1.46 | -64.1±1.87 | *0.61* |
| Rheobase (pA) | 161.7±19.39 | 110.5±13.66 | **0.048* |
| Current threshold (mV) | -39.5±1.27 | -42.1±0.84 | *0.09* |
| Input resistance (Mohm) | 124.4±13.93 | 141.7±9.54 | *0.30* |
| Membrane capacitance (pF) | 158.5±9.04 | 149.8±8.23 | *0.48* |
| Sag ratio | 0.12±0.017 | 0.12 ±0.012 | *0.89* |
| Time constant (ms) | 21.7±1.86 | 26.8±2.32 | *0.10* |
| **Active properties** | | | |
| AP amplitude (mV) | 84.8±4.23 | 79.1±2.09 | *0.52* |
| AP half-width (msec) | 1.26±0.096 | 1.50±0.054 | **0.002* |
| AHP (mV) | -13.5±0.78 | -13.2±0.86 | *0.81* |
| AHP time to peak (ms) | 42.6±3.55 | 45.1±3.47 | *0.62* |
| dv/dtmin (mV/ms) | -58.3±5.18 | -49.1±2.55 | *0.12* |
| dv/dtmax (mV/ms) | 258.9±24.10 | 215.9±12.05 | *0.10* |
| Interspike interval (ms) | 47.6±2.44 | 52.3±2.03 | *0.14* |
| Adaptation ratio | 0.39±0.046 | 0.49±0.032 | *0.07* |

| **Supplementary Table 1C. Passive and active membrane properties of PV interneurons in the mPFC at 20 weeks** | | | |
| --- | --- | --- | --- |
|  | **PV interneurons** | | |
|  | **PV-Cre-tdTomato** | **APP/PS1-PV-Cre-tdTomato** | ***p-value*** |
| **Properties** | (mean ± SEM) | (mean ± SEM) | (Student’s *t*-test) |
| **Passive properties** | | | |
| RMP (mV) | -70.22±0.65 | -71.2±0.75 | *0.36* |
| Rheobase (pA) | 307.4±16.18 | 265.4±19.17 | *0.10* |
| Current threshold (mV) | -34.7±0.70 | -36.6±1.24 | *0.17* |
| Input resistance (Mohm) | 83.7±3.16 | 89.6±4.49 | *0.28* |
| Membrane capacitance (pF) | 64.8±3.36 | 68.1±5.67 | *0.61* |
| Sag ratio | 0.10±0.0051 | 0.10±0.0057 | *0.57* |
| Time constant (ms) | 6.1±0.27 | 6.2±0.32 | *0.63* |
| **Active properties** | | | |
| AP amplitude (mV) | 59.1±1.42 | 59.1±1.43 | *0.99* |
| AP half-width (msec) | 0.46±0.011 | 0.46±0.014 | *0.71* |
| AHP (mV) | -19.2±0.37 | -18.6±0.43 | *0.31* |
| AHP time to peak (ms) | 1.37±0.047 | 1.39±0.056 | *0.76* |
| dv/dtmin (mV/ms) | -158.9±7.45 | -157.1±8.32 | *0.87* |
| dv/dtmax (mV/ms) | 243.7±8.35 | 239.8±9.04 | *0.76* |
| Interspike interval (ms) | 23.2±1.73 | 21.9±1.37 | *0.61* |
| Adaptation ratio | 1.30±0.318 | 1.04±0.050 | *0.58* |

| **Supplementary Table 1D. Passive and active membrane properties of PYR neurons in the mPFC at 20 weeks** | | | |
| --- | --- | --- | --- |
|  | **PYR neurons** | | |
|  | **PV-Cre** | **APP/PS1-PV-Cre** | ***p-value*** |
| **Properties** | (mean ± SEM) | (mean ± SEM) | (Student’s *t*-test) |
| **Passive properties** | | | |
| RMP (mV) | -67.3±0.95 | -65.9±1.09 | *0.36* |
| Rheobase (pA) | 130.2±8.85 | 104.3±6.79 | **0.022* |
| Current threshold (mV) | -40.4±0.54 | -40.8±0.53 | *0.64* |
| Input resistance (Mohm) | 125.6±8.64 | 136.0±9.63 | *0.48* |
| Membrane capacitance (pF) | 163.3±9.11 | 167.0±9.19 | *0.78* |
| Sag ratio | 0.14±0.013 | 0.13±0.009 | *0.94* |
| Time constant (ms) | 23.2±1.40 | 24.8±1.10 | *0.39* |
| **Active properties** | | | |
| AP amplitude (mV) | 84.1±1.75 | 84.7±1.03 | *0.79* |
| AP half-width (msec) | 1.25±0.035 | 1.17±0.024 | *0.16* |
| AHP (mV) | -13.0±0.47 | -12.9±0.42 | *0.84* |
| AHP time to peak (ms) | 34.7±1.99 | 34.8±1.38 | *0.98* |
| dv/dtmin (mV/ms) | -58.4±2.23 | -63.1±1.66 | *0.10* |
| dv/dtmax (mV/ms) | 263.2±11.57 | 267.4±7.43 | *0.76* |
| Interspike interval (ms) | 69.7±1.20 | 69.0±1.10 | *0.67* |
| Adaptation ratio | 0.50±0.027 | 0.44±0.023 | *0.09* |

| **Supplementary Table 2A. Passive and active membrane properties of SST interneurons in the mPFC at 20 weeks** | | | |
| --- | --- | --- | --- |
|  | **SST interneurons** | | |
|  | **PV-Cre** | **APP/PS1-PV-Cre** | ***p-value*** |
| **Properties** | (mean ± SEM) | (mean ± SEM) | (Student’s *t*-test) |
| **Passive properties** | | | |
| RMP (mV) | -61.2±2.14 | -58.7±1.22 | *0.47* |
| Rheobase (pA) | 76.2±6.50 | 75.4±8.20 | *0.75* |
| Current threshold (mV) | -41.9±1.24 | -42.2±1.09 | *0.86* |
| Input resistance (Mohm) | 200.2±15.03 | 202.0±11.74 | *0.93* |
| Membrane capacitance (pF) | 98.4±6.33 | 86.6±6.14 | *0.20* |
| Sag ratio | 0.16±0.012 | 0.16±0.018 | *0.9* |
| Time constant (ms) | 21.7±2.09 | 20.9±1.75 | *0.77* |
| **Active properties** | | | |
| AP amplitude (mV) | 72.3±2.16 | 69.5±1.90 | *0.34* |
| AP half-width (msec) | 0.74±0.04 | 0.75±0.06 | *0.83* |
| AHP (mV) | -13.1±1.31 | -14.3±1.54 | *0.55* |
| AHP time to peak (ms) | 2.1±0.15 | 2.1±0.23 | *0.78* |
| dv/dtmin (mV/ms) | -113.8±9.41 | -114.0±10.56 | *0.99* |
| dv/dtmax (mV/ms) | 204.5±13.56 | 187.3±13.01 | *0.37* |
| Interspike interval (ms) | 53.0±4.09 | 43.4±3.7 | *0.18* |
| Adaptation ratio | 0.57±0.04 | 0.61±0.04 | *0.48* |
